# Evolution of Chikungunya virus in mosquito cells

**DOI:** 10.1038/s41598-018-34561-x

**Published:** 2018-11-01

**Authors:** Souand Mohamed Ali, Abdennour Amroun, Xavier de Lamballerie, Antoine Nougairède

**Affiliations:** Unité des virus Émergents (UVE; Aix-Marseille Univ – IRD 190 – Inserm 1207 – IHU Méditerranée Infection), Marseille, France

## Abstract

It has been observed that replication of Chikungunya virus (CHIKV) in C6/36 *Aedes albopictus* cells has little effect on virus evolution. To characterize evolutionary patterns associated with CHIKV replication in mosquito cells, we performed serial passages of the LR2006 strain in *Ae. albopictus* cells (75 and 30 passages in C6/36 and U4.4 respectively) and *Ae. aegypti* cells (100 passages in AA-A20 and in AE) and studied genotypic changes accompanying adaptation during this evolutionary process. Quantitative analysis revealed cell specific patterns: low mutation rates in C6/36 cells except when a CHIKV strain pre-adapted to mammalian was used and typical features of adaptation to cell culture conditions with a high number of fixed mutations in AE and AA-A20 cells probably due to the weak permissiveness of these latter cell lines. Altogether, these results suggested that both cell line and viral strain influence rates of viral evolution. In contrast, characteristics and distribution of mutations were qualitatively very similar in all mosquito cells with a high level of parallel evolution including 4 deletion mutations. Serial passage in mammalian cells of viruses pre-adapted to mosquito cells revealed disappearance of almost all shared mutations suggesting that many of these mutational patterns are vector-specific.

## Introduction

RNA viruses are characterized by high mutation rates^1–3^. Mutations are frequently incorporated during viral RNA replication due to low fidelity of the viral RNA dependent RNA polymerase (RdRP) and the inability to correct errors^4^. Therefore, the continuous generation of intra-population genetic diversity results in genetic plasticity and consequently high adaptability of RNA viruses^1,5^.

Almost all arthropod-borne viruses (arboviruses) are single stranded RNA viruses. These infectious agents evolve more slowly than other RNA viruses in nature. This genetic stability is believed to result from the requirement of these viruses to be able to replicate in vertebrate and arthropod hosts, each of which imposes specific selective pressures. The adaptation for optimal fitness in either host type involves a trade-off for fitness in the other host^4,6–9^.

Substantial previous studies have already been carried out to understand mechanisms of fitness trade-off and, in most cases, a similar experimental design was employed^10–18^. Arboviruses were serially passaged either in vertebrate or arthropod cells or in each cell line alternately to simulate the natural cycle of the virus and the fitness of progeny viruses was assessed relative to progenitors. These studies revealed general patterns of arbovirus evolution: (i) most of the time, adaptation of the virus to a single host resulted in a fitness gain in the same environment^18^, (ii) observation of fitness trade-offs (*i.e*. adaption to vector/host cells resulting in loss of fitness in the bypassed host/vector cells) was unpredictable and sometimes fitness trade-offs were asymmetrical^10–17^, (iii) alternation between vector and host cells generally resulted in fitness increases in one or both vector/host cells^10–17^.

In some of these experimental evolution studies, the genotypic changes accompanying the fitness modifications were also studied. Sometimes, alternation between host and vector cells resulted in evolutionary stasis in accordance with the trade-off theory^10,11,14,16^ but in many other studies, host alternation induced the emergence of mutations as observed when the virus evolved in one unique cell type^19^. These studies also delineated the characteristics of the mutations. Most were non-synonymous, contrasting with the large majority of synonymous mutations observed in nature and revealing strong purifying selection during the natural cycle of these viruses. Their distribution in the viral genome greatly differed according to the virus and the experimental conditions used, some studies found mutational hot spots located in genes encoding non-structural proteins^10,14^, but others were found in genes encoding structural proteins^15,19^.

When working with arboviruses transmitted by mosquitoes such as dengue virus, eastern equine encephalitis virus, Sindbis virus or chikungunya virus (CHIKV), it was often observed that the rate of mutation accumulation was slower when viruses were serially passaged in mosquito cells suggesting that replication in mosquito cells has little effect on virus evolution^10–12,14,15^. In almost all these studies only the C6/36 *Aedes albopictus* cell line was used. These highly permissive cells were initially selected to isolate and cultivate arboviruses and recent studies demonstrated that the RNA interference pathway, a critical aspect of the cellular innate antiviral immune response in invertebrates, does not function properly in C6/36 cells^20,21^. Measuring rates of mutation accumulation in other mosquito cells could help to clarify the particular effect of using C6/36 cells on virus evolution.

CHIKV is a small, enveloped, single-stranded positive-sense RNA virus with a genome of approximately 12 kb that contains two open reading frames (ORFs) encoding non-structural and structural proteins, respectively. In the sylvatic environment this arbovirus, transmitted by *Aedes* species mosquitoes, circulates in an enzootic cycle involving non-peridomestic mosquitoes and non-human primates in Africa and Asia. CHIKV also causes explosive urban outbreaks of febrile arthralgia associated with a “human-mosquito-human” transmission cycle involving *Ae. aegypti* and more recently *Ae.* *albopictus* mosquitoes^9,22,23^. This virus is an excellent example of a re-emerging pathogen. It recently spread throughout large regions of the American continent and the presence of the competent vector *Ae. albopictus* in temperate regions raises the realistic possibility of its expansion in Europe and northern Asia^24–27^.

The main objective of this work was to conduct a comprehensive study on arbovirus evolution in mosquito cells to characterize cell-specific evolutionary patterns and mutational patterns of adaptation to mosquito cells. Using the LR2006 CHIKV strain that belongs to the East-Central-South-African (ECSA) genotype as a model, we performed serial passages in *Ae. albopictus* (C6/36 and U4.4) and *Ae. aegypti* (AA-A20 and AE) cell lines^28^. We focused almost exclusively on the genotypic changes accompanying adaptation during experimental evolution.

## Materials and Methods

### Cells

*Ae. aegypti* (AA-A20 and AE) and *Ae. albopictus* (C6/36 and U4.4) cells were maintained in L-15 medium (Life Technologies) with 10% fetal bovine serum (FBS), 1% Penicillin/Streptomycin (PS; 5000 U/ml and 5000 µg/ml; Life technologies) and 1% tryptose phosphate (29.5 g/L; Sigma-Aldrich) at 30 °C. African green monkey cells (Vero) cells were maintained in Minimal Essential medium (MEM; Life Technologies) with 10% FBS, 1% P/S at 37 °C with 5% CO2.

### Virus

All experiments using replicating viruses were performed in BSL3 facilities.

We used a previously described infectious clone (IC) derived from the LR2006 strain (GenBank accession number EU224268) to produce the virus^15^. The IC was transfected into a 75 cm^2^ culture flask of subconfluent Vero cells (Fugene 6 transfection reagent; Roche). Cells were incubated for 4 hours, washed twice (HBSS; Life technologies) and 20 ml of medium was added. After incubation at 37 °C for three days, supernatant medium was harvested, clarified by centrifugation, aliquoted and stored at −80 °C. The virus was passaged once in Vero cells (called first passage in the study) at an MOI of 0.5: a 175 cm^2^ culture flask of confluent Vero cells was infected for 2 hours with the virus diluted appropriately. The cells were then washed twice (HBSS) and incubated for 48 hours at 37 °C following the addition of 50 ml of culture medium. Supernatant medium was harvested, clarified by centrifugation, aliquoted and stored at −80 °C (virus stock).

### Serial passage of CHIKV *in cellulo*

All experimental studies of the effects of serial passage were initiated using the same virus stock (the first passage, see above). Among the passages reported in this study, 50 in C6/36 (C6/36-1 virus) were previously described^15^. Other serial virus passages were performed over a period of several years which explains why the number of passages (total number of passages and the number of passages used for sequencing) as well as the sequencing methods are not always the same. Nevertheless, achievement of serial virus passage in successive phases ensured effective prevention of cross contamination. In situations when viruses were passaged at overlapping times, the manipulations were always sequential and carried out in different biological safety cabinets.

Except for the first ten passages in *Ae. aegypti* cells (see below), the same general procedure was used to perform all serial passages. Diluted clarified infectious cell supernatant medium was used to infect for 2 hours a 25 cm^2^ a culture flask of confluent cells. At each passage, an estimated MOI of approximately 0.1 was used. Cells were washed (HBSS) and 7 mL of medium was added before incubation. Supernatant medium was harvested at 48 hours post-infection, clarified by centrifugation, aliquoted and stored at −80 °C.

Following many unsuccessful attempts to produce infectious virus in *Ae. aegypti* cells, we decided to perform the serial passages with undiluted supernatant medium from viruses previously passaged in C6/36 cells for the first ten passages. Undiluted clarified supernatant medium was used to infect for 2 hours a 25 cm² culture flask of confluent cells. Cells were washed (HBSS) and 7 mL of medium was added before incubation at 37 °C. Supernatant medium was harvested at 48 hours post-infection, clarified by centrifugation, aliquoted and stored at −80 °C. The C6/36-1 virus at passages 18 and 31 were finally used as the starting point to perform the first ten passages in AE and AA-A20 cells respectively. The subsequent 40 passages in *Ae. aegypti* cells were performed following the general procedure described above.

### Complete genome amplification by RT-PCR

To avoid DNA contamination, viral genome amplifications were performed in a molecular biology laboratory dedicated to clinical diagnosis which includes specific laboratories for each step of the process: nucleic acid extraction, mix preparation, RNA/cDNA manipulation, amplification and PCR product manipulation. In addition, complete genome amplification carried out at the same time was always performed in separate experiments.

Viral RNA was extracted from clarified supernatant medium using the EZ1 Virus Mini Kit v2 on the EZ1 Advanced XL Biorobot (both from Qiagen). Two complete genome amplification procedures were employed based on the sequencing method used. When Sanger sequencing was performed, a specific set of primers was used to generate amplicons covering the entire genome (excluding the first 18 nucleotides of the 5′UTR and the 22 nucleotides upstream of the polyA tail) with the Access RT-PCR System (Promega) as previously described^15^. When Next-generation Sequencing (NGS) was performed, the complete viral genomes (excluding the first 18 nucleotides of the 5′UTR and the 88 nucleotides upstream of the polyA tail) were amplified in four fragments using specific sets of primers (Supplemental Table 6 in Supplemental Data) with the Superscript III One-Step RT-PCR Platinum TaqHifi kit (Life Technologies) following manufacturer’s instructions.

### Sanger sequencing

Purified PCR products were sequenced with both forward and reverse primers using the BigDye Terminator v3.1 Cycle Sequencing Kit on an ABI Prism 31310X Genetic Analyser sequencer (both from Applied Biosystems). Analysis of sequencing chromatogram and combination of sequences was performed using the Sequencher 5.0 software (Gene Codes Corporation). Using the sequence of the original virus (IC viral sequence) as reference, complete viral genome sequences were constructed. We took into account the fixation rate of each mutation detected by associating a quantitative value to each of them: 1 when one peak was detected on the sequencing chromatogram (fixed mutation), 0.75 or 0.25 when two peaks of different intensity were detected (0.75 or 0.25 for majority or minority mutations respectively) and 0.5 when two peaks of comparable intensity were detected (Supplemental Table 4 in Supplemental Data).

### Next-Generation sequencing

Next-Generation Sequencing (NGS) was performed using the Ion PGM Sequencer (Life Technologies) and analysis of sequencing data were conducted with the CLC Genomics Workbench 6 software (CLC Bio). For each virus passage sequenced, purified PCR products were pooled and analyzed using the Ion PGM Sequencer according to the manufacturer’s instructions. The read sequences obtained were trimmed, first using quality score, then by removing the primers used during the RT-PCR and finally at the 5′ and 3′ termini by systematically removing 6 nt. Only reads with a length greater than 29 nt were used and mapped for comparison to the sequence of the original virus (IC viral sequence). A minimum coverage of 1000 was obtained for all complete genome sequences analyzed. Mutation frequency (proportion of viral genomes with the mutation) for each position was calculated as the number of reads with a mutation divided by the total number of reads at that site. Only substitutions with a mutation frequency ≥2% have been used for further analysis (Supplemental Table 4 in Supplemental Data).

### Genome sequence analysis

The analysis was conducted on mutations covering the entire viral genome excluding the first 18 nucleotides of the 5′UTR and the 88 nucleotides upstream of the polyA tail. For NGS sequencing data, only mutations detected with a mutation frequency of at least 20% were considered when Sanger and NGS data were used together to compare the number of mutations (Supplemental Table 5 in Supplemental Data summarized all the sequencing data analyzed in this study).

Complete genome sequence of CHIKVs (n = 166) were manually extracted from Genbank. Nucleotide sequences of the two ORFs were manually extracted, concatenated and aligned according to the amino acid sequence using Mega 6 software^29^. Ambiguously aligned regions were corrected manually. To avoid artifacts, high passage strains as well as strains potentially contaminated were removed from the final alignment as previously described^30^. Because most sequences belonged to recent epidemic lineages, we used Phylogenetic Diversity Analyser software to select a representative subset of sequences based on a phylogenetic tree^31^.

Based on phylogenetic analysis (maximum likelihood method using Mega 6 software), we used sequences belonged to the ECSA lineage (n = 49; Supplemental Note 1 in Supplemental Data) to determine the variability 1^st^ + 2^nd^ and 3^rd^ codon positions.

To compare shared mutations found in our experiments with variable sites reported in Genbank ECSA CHIKV genomic sequence, the number of common variable sites between the Genbank sequences and our shared substitution was recorded. The number of common sites expected under a regimen of random distribution was calculated by dividing the product (common variable site at 3rd codon position detected in Genbank sequence multiplied by synonymous shared substitution observed in our experiment) by the total number of nucleotides at the 3rd codon position. To describe this distribution statistically, a binomial distribution with parameters (n: number of common sites, p: length of the sequence) (R online software) was used.

### Real time RT-PCR assay

Viral RNA was extracted from clarified supernatant medium as described above. The amount of viral RNA was determined using a quantitative real-time RT-PCR assay (GoTaq 1-Step RT-qPCR System, Promega) as described previously^15^. The mixture (final volume: 20 μL) contained 10 μL of Master Mix 2X Reaction Buffer, 0.5 μM of each primer, 0.3 μL of probe, 0.5 μL of GoSCRIPT RT Mix, 3.2 μL of Nuclease-Free Water and 5 μL of extracted nucleic acids. Sequences of primers/probes are detailed in Supplemental Table 7 in Supplemental Data. Assays were performed on CFX96 thermocycler (Biorad) under standard amplification conditions with a 60 °C hybridization temperature. The quantity of viral RNA (number of genome copies per mL) was calculated using standard curves produced from serial dilutions of nucleic acids.

### Infectious titres estimated using a TCID50 assay

TCID50 titrations were performed with Vero cells as described previously^15^. Briefly, microtitre culture plates (96 wells) containing cell monolayers were inoculated with serial 10-fold dilutions of clarified infectious supernatant medium, incubated for 7 days and read for absence or presence of cytopathic effect in each well. The determination of the TCID50/mL was performed using the method of Reed and Muench.

## Results

### Serial passage of virus in mosquito cells

**Figure 1:**
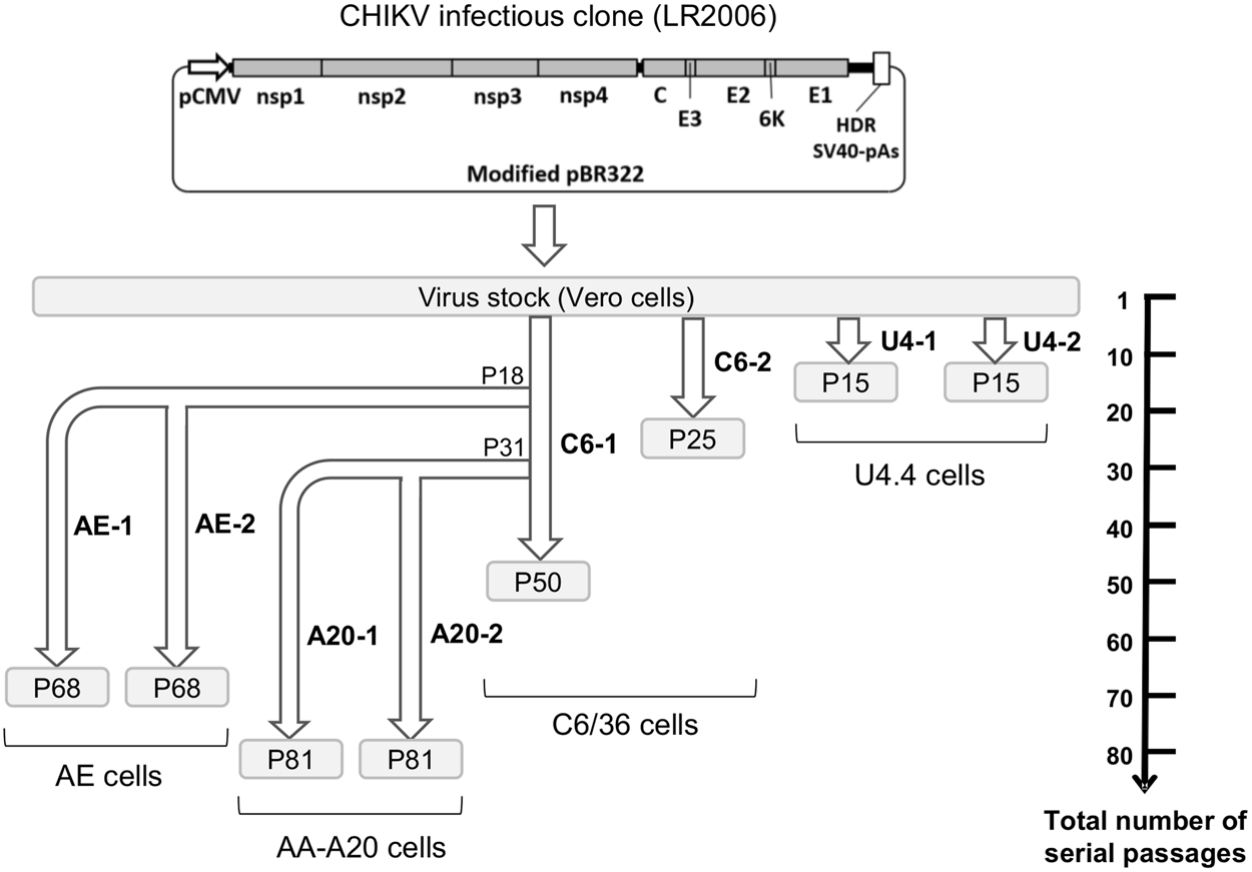
Schematic representation of experimental strategy used in this study. The parental strain derived from an infectious clone (LR 2006 strain). Virus stock was obtained following one passage in Vero cells and was used to perform serial passages in C6/36 (named C6-1 and C6-2) and U4.4 (named U4-1 and U4-2) cells. Viruses serially passaged 18 and 31 times in C6/36 cells were used to perform passages in AE cells (designated AE-1 and AE-2) and AA-A20 (designated A20-1 and A20-2) cells respectively.

The parental virus strain was derived following transfection of a previously described CHIKV IC into Vero cells^15^ (LR2006 strain). Virus stock was then obtained following one passage in Vero cells (Fig. 1).

A general procedure was chosen to perform serial passages of the virus in cell cultures. An estimated MOI of approximatively 0.1 was used to infect cells and each passage was terminated after 48 hours (~8 replication cycles per passage)^15^. This general procedure was successfully applied to perform passages in *Ae. albopictus* cells. Two viruses were independently serially passaged in C6/36 (50 and 25 passages; named C6-1 and C6-2) and in U4.4 cells (15 passages for both duplicates; named U4-1 and U4-2) (Fig. 1). During these serial passages, viral RNA yields in supernatant medium fluctuated between 6.8 × 10^9^ and 9.1 × 10^11^ copies/mL.

**Figure 2:**
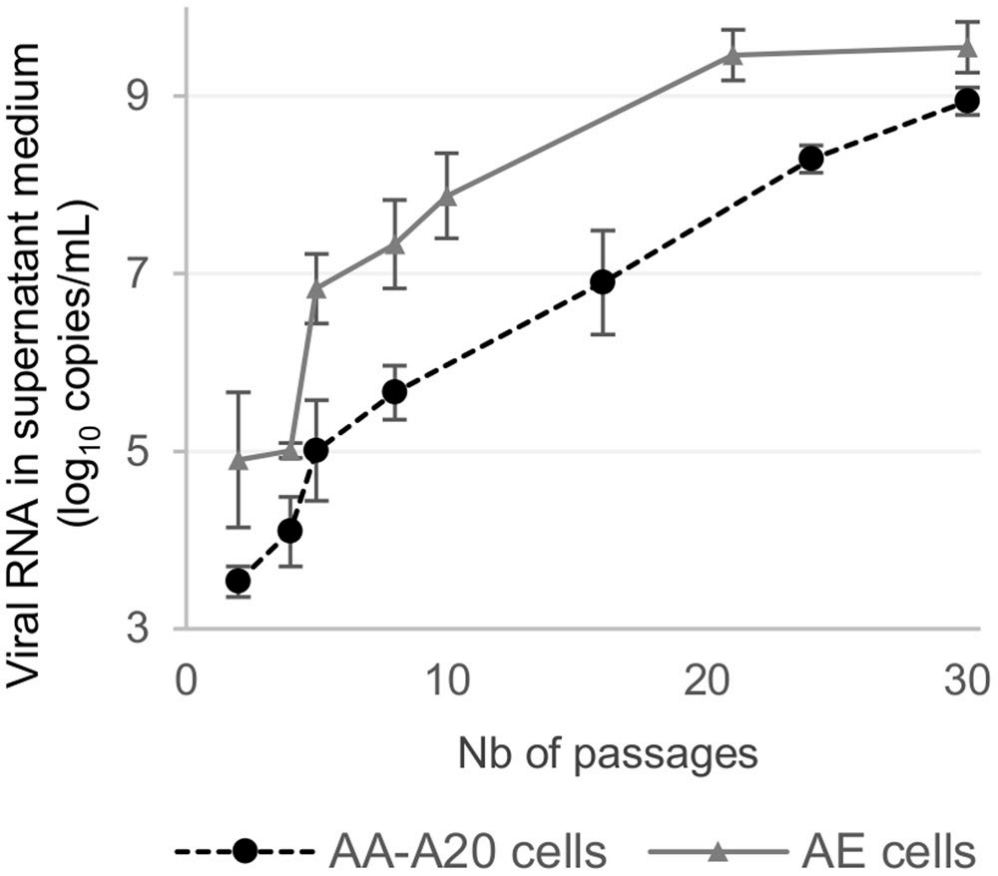
Evolution of viral production during the first 30 serial passages in *Ae. Aegypti* cells.

We failed to produce infectious virus in *Ae. aegypti* cells (AE and AA-A20 cells) using the procedure described above. Therefore, we performed the serial passages with undiluted supernatant medium from viruses previously passaged in C6/36 cells. Using this approach, we successfully passaged, 10 times, viruses in duplicate in AE and AA-A20 cells using as the starting point the C6-1 virus respectively at passages 18 and 31 times in C6/36 cells (named AE-1 and AE-2; A20-1 and A20-2) (Fig. 1). During these passages, we observed a constant increase in the amount of viral RNA in supernatant media suggesting that the viruses had progressively increased their replicative fitness (Fig. 2). The subsequent 40 serial passages were performed following the same procedure as described for viruses passaged in *Ae. albopictus* cells.

### Genome sequence analysis of passaged viruses

Because virus passages were performed over a period of several years, passages used for sequencing as well as sequencing methods (Sanger or NGS) were not always identical. For each virus-containing supernatant medium analyzed, the complete genome sequence was established. For each mutation detected, its frequency (*i.e*., fixation rate) was calculated or estimated when NGS or Sanger sequencing was performed respectively (see Materials and Methods section).

#### Emergence of substitutions during serial passage of the viruses

All viruses serially passaged in insect cells exhibited appearance of mutations when substitutions with a frequency ≥20% were considered (allowing unbiased comparison between sequencing data generated using NGS and Sanger sequencing methods) (Supplemental Fig. 1A in Supplemental Data). However, the average number of substitutions observed in viruses passaged in C6/36 cells reached a plateau after 30 passages while viruses passaged in AA-A20 and AE cells exhibited a continuous appearance of substitutions (Supplemental Fig. 1A in Supplemental Data). Consequently, the average number of substitutions with a frequency ≥20% observed during 50 passages in C6/36 (n = 4) was one half of that observed during 50 passages in AA-A20 and AE cells (n = 8.5).

When only mutations fixed or almost fixed were considered (substitutions with a frequency ≥75%), two different evolutionary pathways were observed according to the cells used during serial passage (Supplemental Fig. 1B in Supplemental Data): the maximum value of the average number of fixed substitutions was very low in C6/36 and U4.4 cells (max value = 1) while this value was 3.5 and 5.5 during passages in AA-A20 and AE cells respectively.

**Figure 3:**
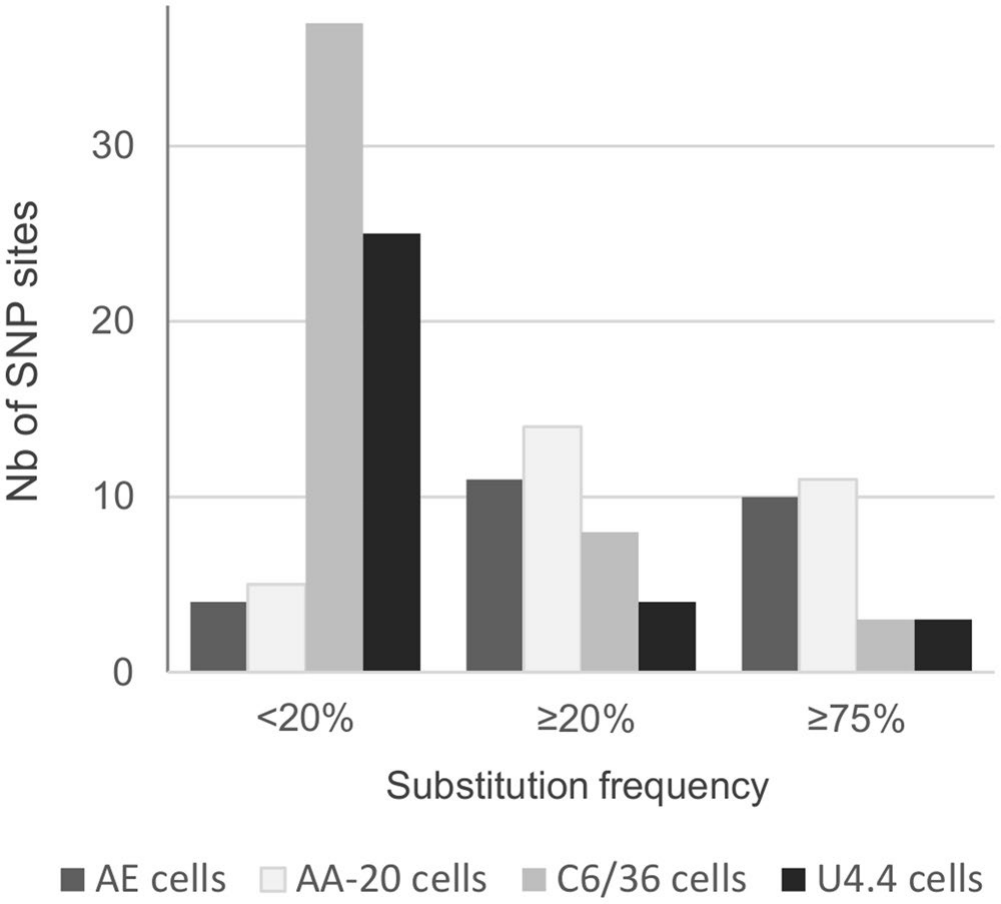
Number of single nucleotide polymorphic (SNP) sites detected per cell line. Each variable nucleotide position (SNP site) was counted once. To allow unbiased comparison, we take into account only 50 serial passages from the parental strain except for U4.4 cells for which only 15 passages were performed in duplicate. The maximum frequency was considered when one substitution was detected more than one time.

In accordance with the results presented above, the study of the number of single nucleotide polymorphic (SNP) sites detected in each cell line revealed a higher global genomic variability during serial passage in AA-A20 and AE cells than in C6/36 and U4.4 cells (Fig. 3; 11–14 versus 8–4 SNP sites when substitutions with a frequency ≥20% considered and 10–11 versus 3 SNP sites when substitutions with a frequency ≥75% were considered). Interestingly, when considering only minority variants (only substitutions with a frequency ≤20% were counted), a higher variability was observed in C6/36 and U4.4 cells (Fig. 3; 25–37 versus 4).

All together, these findings demonstrated that replication in mosquito cells can have various effects on virus evolution depending on the cell line used. Nevertheless, in accordance with previous studies we observed that replication in C6/36 cells has little effect on virus evolution^10,11,14,15^. This observation may be related to the intrinsic properties of this mosquito cell line and/or the phenotype of the viral strain used. To determine the role of the viral strain, we performed serial passages in C6/36 cells of the same virus (identical parental strain) already adapted to mammalian cells. The strain was serially passaged 80 times in Vero cells and then 20 times in duplicate in C6/36 cells. Using the complete genome sequence of the virus passaged 80 times in Vero cells as reference, emergence of substitutions after 20 passages was studied. In comparison with the low number of mutations observed during serial passages in C6/36 (a maximum of 4 and 1 mutations was observed when substitutions with a frequency ≥20% or ≥75% were considered respectively), we found a high number of mutations (Supplemental Table 1 in Supplemental Data; 8–13 when substitutions with a frequency ≥20% were considered; 5 when substitutions with a frequency ≥75% were considered). These findings demonstrate that the phenotype of the viral strain has a significant effect on virus evolution in C6/36 cells.

Of note, 8 substitutions that appeared during serial passage in mosquito cells, completely disappeared following subsequent passages (Supplemental Table 2 in Supplemental Data). Seven of these transitory mutations were fixed (mutation frequency ≥75%). Interestingly, all of these mutations occurred during the passages in AA-A20 and AE cells.

#### Characteristics and distribution of substitution detected during serial passage of the viruses

In all mosquito cell lines, we detected a majority of non-synonymous substitutions (ranging from 54% in AE cells to 66% in U4.4 cells; Supplemental Fig. 2A in Supplemental Data). The proportion of synonymous substitutions is slightly higher when only minority variants (mutation frequency ≤20%) were considered (29–50% versus 0–29% when compared to substitutions with a frequency ≥20%; Supplemental Fig. 1B,C in Supplemental Data**)**. Except for the viruses passaged in U4.4 cells, a high proportion of substitutions was also detected in the untranslated regions which represent only 5.4% of the complete genome (ranging from 13% in AA-A20 cells to 23% in AE cells; Supplemental Fig. 2A in Supplemental Data). The original sequence of the LR2006 did not contain an opal codon at the 3′ extremity of the nsP3 coding region and we did not observed appearance of this opal codon during all the serial passages in mosquito cells^32^.

**Figure 4:**
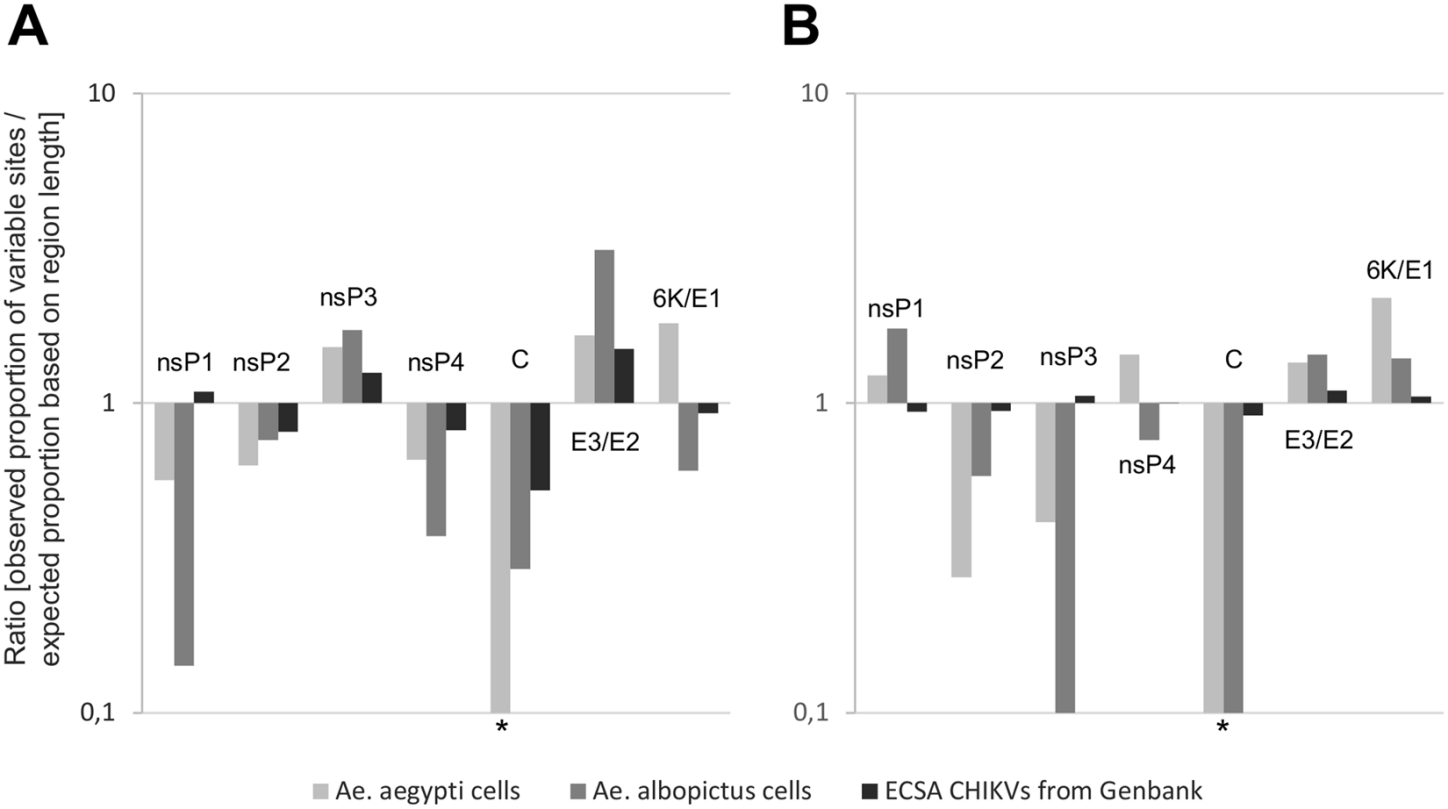
Substitution distributions on coding regions. (**A**) Non-synonymous substitutions for viruses passaged in *Ae. aegypti* or *albopictus* cells, and variability at 1st + 2nd codon positions for ECSA CHIKVs from GenBank were taken into account. (**B**) Synonymous substitutions for viruses passaged in *Ae. aegypti* or *albopictus* cells, and variability at 3rd codon position for ECSA CHIKVs from GenBank were taken into account. Each variable nucleotide position (SNP site) was counted only once per group (*i.e. Ae. aegypti*, *albopictus* cells or ECSA CHIKVs from GenBank). For a given region, the observed proportion of variable sites corresponds to the number of variable sites in this region divided by the total number of variables sites. This proportion was divided by an expected proportion based on region length (length of the region divided by the total length of coding regions). *A value of 0.1 was used when no variable site was found.

The distribution of the non-synonymous substitutions over the different coding regions of the genome was not random and was very similar in *Ae. aegypti* and *Ae. albopictus* cells (Fig. 4A). In particular, nsP3 and E3/E2 regions were highly variable and contained respectively 22% and 32% of the synonymous variable sites while these regions represent respectively only 14% and 13% of the complete coding regions of the viral genome. In contrast, the nsP1, nsP4 and C regions varied very little and contained respectively 5%, 9% and 1% of the synonymous variable sites while these regions represent respectively 14%, 16% and 7% of the complete coding regions of the viral genome. The distribution of the non-synonymous mutations matched that of genetic variability observed in CHIKV genomes retrieved from GenBank (1^st^ and 2^nd^ codon positions; Fig. 4A) except in the nsP1 region.

Of note, the reversion of the *Ae. albopictus*-adaptative mutation E1-A226V was observed during all serial passages in mosquito cells.

The distribution of the synonymous substitutions over the different coding regions of the genome was also not random (Fig. 4B). In particular, nsP1, E3/E2 and 6 K/E1 regions were highly variable and contained respectively 21%, 18% and 24% of the synonymous variable sites while these regions represent respectively only 14%, 13% and 13% of the complete coding regions of the viral genome. In contrast, the nsP2, nsP3 and C regions varied very little and contained respectively 9%, 9% and 0% of the synonymous variable sites while these regions represent respectively 21%, 14% and 7% of the complete coding regions of the viral genome. The genetic variability observed in CHIKV genomes retrieved from GenBank (3^nd^ codon positions; Fig. 4B) is almost random and does not match with the distribution of the synonymous mutations observed during serial passage in mosquito cells.

**Figure 5:**
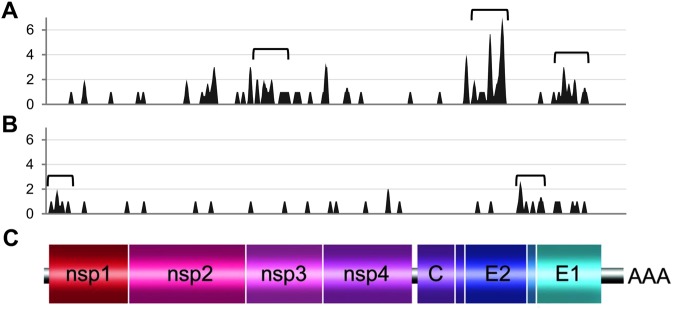
Patterns of substitution distributions on coding region. (**A)** Non-synonymous substitutions. (**B)** Synonymous substitutions. (**C)** Schematic representation of the CHIKV complete genome. Each variable nucleotide position (SNP site) was counted only once. The variability was represented using 75 nt sliding windows. Brackets indicate mutational hot spot (defined for each nt position as 450 nt sliding window interval containing more than 8 or 4 variable sites for non-synonymous or synonymous substitutions respectively).

Study of patterns of mutation distributions along the genome (number of variable nucleotide positions per sliding window interval of 75 nt; Fig. 5) confirmed that non-synonymous and synonymous mutations were non-randomly distributed and revealed the presence of specific mutational hot spots. Hot spots of non-synonymous mutations were located in nsP3, E2 and E1 regions while those of synonymous mutations were located in the nsP1 region and in a zone that straddled the E2, 6K, E1 regions (Fig. 5).

#### Parallel evolution in insect cells

Our experiments revealed frequent parallel evolution; a total of 42 substitutions were shared within at least two independent experiments out of a total of 127 mutations detected (each variable nucleotide position was counted only once; Table 1). The characteristics and distribution of these shared mutations were nearly the same as those described above: (i) a majority of non-synonymous substitutions (60%; 25/42), (ii) 12% (5/42) of these located in untranslated regions and (iii) 48% (12/25) of non-synonymous substitutions located in nsP3 and E3/E2 regions.

**Table 1:** Shared substitutions detected during serial passage in mosquito cells.

Nucleotide position	Region	Nucleotide change	AA change	Viruses harbouring the mutation	
				substitution frequency ≥20%	substitution frequency <20%
22	5′UTR	A → U	—	AE-1, AE-2, A20-1, C6-1	—
202	nsP1	G → A	—	AE-1, AE-2	—
226	nsP1	A → G	—	—	AE-1, AE-2
442	nsP1	A → G	—	A20-1	A20-2
782	nsP1	U → C	C → R	AE-1, AE-2	—
1320	nsP1	U → C	L → P	A20-1, A20-2	—
1976	nsP2	G → U	V → L	A20-1, A20-2	—
3040	nsP2	G → A	—	—	AE-1, AE-2
3402	nsP2	U → C	F → S	—	A20-1, A20-2
3440	nsP2	G → A	V → M	AE-1, AE-2	—
4151	nsP3	C → U	R → C	AE-2, A20-2, U4-1	U4-2
4167	nsP3	G → A	G → D	AE-1, AE-2, A20-1, A20-2	—
4295	nsP3	U → C	S → P	A20-2	U4-2
4587	nsP3	U → C	I → T	A20-1, A20-2, C6-1	U4-1
5160	nsP3	C → U	A → V	A20-1, A20-2	—
5317	nsP3	U → C	—	AE-1, AE-2	—
5776	nsP4	A → U	—	—	A20-1, A20-2
6158	nsP4	A → U	N → Y	AE-1, AE-2	-
6955	nsP4	A → G	—	A20-1	A20-2
6970	nsP4	U → A	—	—	A20-1, A20-2
7189	nsP4	G → U	—	—	C6-1, C6-2, U4-1
7416	nsP4	U → A	M → K	AE-1, AE-2, A20-1, A20-2	—
7502	Junction	G → A	—	A20-2	AE-1, AE-2
7519	Junction	C → A	—	AE-1, AE-2	—
7522	Junction	U → C	—	AE-2	AE-1
8549	E2	A → G	K → R	U4-1	A20-1
8702	E2	U → C	I → T	AE-2, A20-1, A20-2	—
8738	E2	A → C	K → T	A20-1, A20-2	—
8920	E2	C → A	H → N	—	C6-2, AE-2
9019	E2	A → U	T → S	AE-1, A20-1	—
9064	E2	A → G	T → A	—	AE-1, AE-2
9311	E2	U → G	I → S	A20-1, A20-2	U4-1
9777	E2	U → C	—	AE-2, A20-2	—
10339	E1	A → G	T → A	A20-1, A20-2	—
10445	E1	A → G	D → G	—	A20-1, A20-2
10449	E1	U → C	—	—	A20-1, A20-2
10631	E1	U → C	V → A	—	, AE-2, A20-2
10670	E1	U → C	V → A	AE-1, AE-2, A20-1, A20-2, C6-1, C6-2, U4-1, U4-2	—
10719	E1	U → C	—	AE-1	AE-2
10778	E1	C → U	A → V	A20-1, A20-2	—
10962	E1	C → U	—	—	AE-1, A20-2
11667	3′UTR	A → U	—	—	AE-1, AE-2, A20-2

**Figure 6:**
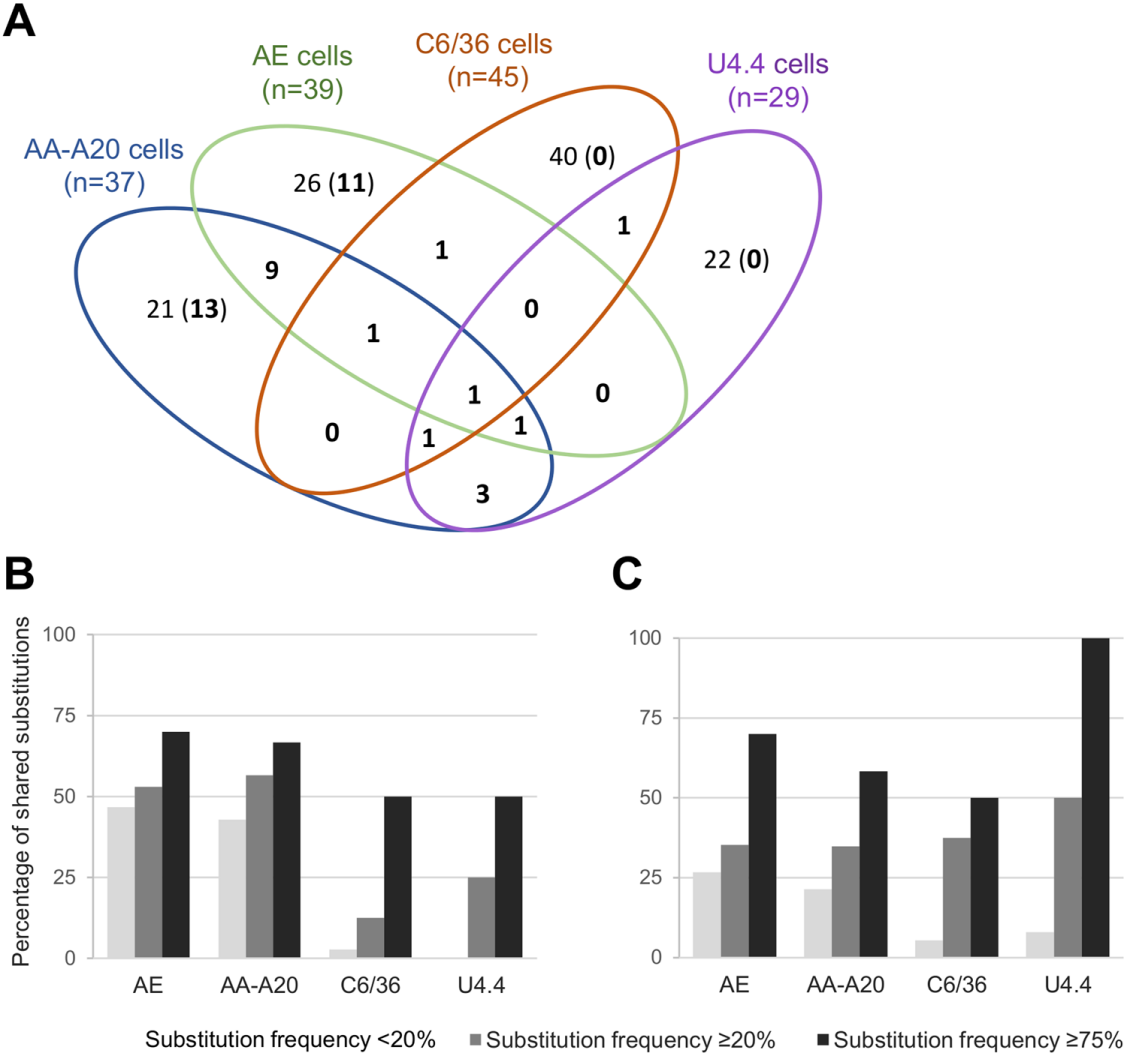
Parallel evolution during serial passage in mosquito cells. (**A**) Venn diagram showing the repartition of the shared substitutions (in bold) during serial passage in mosquito cells. Each cell line is represented by an ellipse. Numbers in overlapping areas represent the number of substitutions found during passages in at least two different cell lines. Numbers in non-overlapping areas represent the number of substitutions found during passages in only one cell line (the number in bracket represents the number of shared substitutions shared in the same cell line). (**B)** Proportion of substitutions shared between viruses passaged in the same cell line. (**C)** Proportion of substitutions shared between viruses passaged in different cell lines (*i.e*. shared between at least two different cell lines). The maximum frequency was considered when one substitution was detected more than once.

Most of this parallel evolution occurred during passaged in both *Ae. aegypti* cell lines (Fig. 6A): 33/42 shared substitutions were found solely in *Ae. aegypti* cells of which 9 were shared between AE and AA-AA20 cells. In contrast, only one shared substitution was found in Ae. albopictus cells only.

Overall, we observed that the higher the frequency of a mutation was, the more this mutation was likely to be shared (Fig. 6B,C). Thus, the proportion of mutations with frequency <20% or ≥75% shared between viruses serially passaged in different cell lines ranged respectively between 8–27% or 50–100% (Fig. 6C). In addition, we observed a higher proportion of low and mid-frequency mutations shared in the same cell line with AE and AA-A20 cell lines (ranging between 43% and 56% versus 0% and 25% in *Ae. albopictus* cell lines; Fig. 6B).

We compared shared mutations found in our experiments with variable sites identified using an alignment of ECSA CHIKV genomes extracted from Genbank. We found that 18% (7/42) of the shared mutations detected were located at variable sites (Table 2). Therefore, a substantial proportion of the shared mutations also varied during the natural life cycle of ECSA CHIKVs. While 60% of the shared substitutions detected were non-synonymous, 100% (7/7) of these common variable sites were synonymous. Because the majority of variable positions were at synonymous sites when comparing ECSA CHIKV genomes extracted from Genbank, we hypothesized that we detect these common mutations by chance. However, we observed that the ratio [observed number of common sites]/[expected number of common sites in case of random distribution] was significantly higher than expected under a regimen of random distribution of variables sites at 3^rd^ codon position (ratio = 2.4; binomial test; *p* = 0.02).

**Table 2:** Shared mutations detected at variable sites of ECSA CHIKV genomes. Variables sites were identified by comparing ECSA CHIKV genomes extracted from Genbank.

Nucleotide position	Region	Nucleotide change	AA change	Viruses harbouring the mutation	
				substitution frequency ≥20%	substitution frequency <20%
202	nsP1	G → A	—	AE-1, AE-2	—
442	nsP1	A → G	—	A20-1	A20-2
5317	nsp3	U → C	—	AE-1, AE-2	—
6970	nsP4	U → A	—	—	A20-1, A20-2
9777	E2	U → C	—	A20-2, AE-2	
10449	E1	U → C	—	—	A20-1, A20-2
10719	E1	U → C	—	AE-1	AE-2

#### Emergence of deletion mutations

Four deletion mutations were found during serial passage in mosquito cells (Table 3). Three were located in regions coding for envelope proteins: one in the E3 region (E3-Phe15del) and two in the E2 region (E2-Val8_Tyr9del and E2-Glu166del). The last mutation was a 15 nt deletion located in the junction region (7512_7526del). Three of these deletion mutations (E3-Phe15del, E2-Glu166del and 7512_7526del) were shared between at least two independent serial passages in mosquito cells and the E2-Val8_Tyr9del mutation was previously detected during several independent serial passages of CHIKVs in C6/36 cells^15^. Interestingly, two of these deletion mutations (E2-Glu166del and 7512_7526del) were fixed during serial passage. Altogether, these results highlight an important role of deletion mutations in adaptation of the virus to mosquito cells.

**Table 3:** Deletion mutations detected during serial passage in mosquito cells. *AA position in the gene (E3 or E2).

Nucleotide position	Region	Nucleotide change	AA position*	AA change	Virus harboring the mutation	
					substitution frequency ≥20%	substitution frequency <20%
7512–26	Junction	Del-CAGCUACCUAUUUUG	—	—	A20-1, A20-2	—
8392–8394	E3	Del-UUC	15	Del-F	—	AE-1, AE-2
8563–8568	E2	Del-GUCUAU	8–9	Del-VY	—	C6-1
9037–9039	E2	Del-GAG	166	Del-E	AE-1, AE-2, A20-1, A20-2, C6-1, U4-1, U4-2	—

#### Specific adaptation to mosquito cells

To determine if shared mutations (including deletion mutations) were related to a specific adaptation to mosquito cells, we selected two viruses (C6–1 passage 31; U4-1 passage 15) that were serially passaged 10 times in duplicate in Vero cells. Before passage in Vero cells, both viruses harbored 5 shared mutations of which 2 or 3 exhibited a frequency ≥20%. After serial passage in Vero cells, all the deletion mutations had completely disappeared as well as a large majority of the substitutions (5/7) (Supplemental Table 3 in Supplemental Data). Of note the remaining 22a → u mutation was previously detected during serial passages in Vero cells indicating that this mutation allows adaptation to both primate and mosquito cells. The other remaining mutation (4587u → c) disappeared completely in one replicate. All these results indicated that the majority of shared mutations are associated with specific adaptation to mosquito cells.

## Discussion

Here we have presented a comprehensive study of arbovirus evolution in mosquito cells. Chikungunya virus derived from a clinical case was serially passaged more than 300 times in *Ae. albopictus* and *Ae. aegypti* cell lines. To determine which genomic changes accompanied adaptation during *in vitro* evolution, the complete genome sequence of these viruses was determined.

A key observation of our study was that viruses passaged in *Ae. albopictus* or *Ae. aegypti* cells followed completely different mutational pathways. In accordance with previous studies, we found that replication in C6/36 cells, and to a lesser extent in U4.4 cells, had little effect on virus evolution with a very low number of mutations fixed during serial passage^10,11,14,15^. In contrast, we found a high number of fixed mutations, transitory mutations, and typical features of adaptation to cell culture conditions (*i.e*. appearance and fixation of a majority of non-synonymous mutations) in AE and AA-A20 cells. This difference may be linked to the fact that we encountered many difficulties to replicate viruses in *Ae. aegypti* cell lines. Indeed, only viruses pre-adapted to C6/36 cell lines were able to reproduce in these cells and viral RNA yield in supernatant medium progressively increased during the first passages while they remained stable during serial passage in *Ae. albopictus* cells. Thus, rates of viral evolution observed during serial passage in cell cultures can be greatly influenced by the initial choice of the cell line.

Our experiments suggest that C6/36 cells are very permissive for replication of CHIKV and/or that the viral CHIKV strain used in this study was already highly adapted to these cells. To test this latter hypothesis, we serially passaged CHIKV in C6/36 cells after a long adaptation to mammalian cells and we observed a strong selection pressure with a high number of mutations fixed during only 20 serial passages. All these results indicated that circulating CHIKVs are highly adapted to C6/36 cells probably because this cell line was originally selected for its high permissiveness to a wide range of arboviruses^21^ and it is now known that RNA interference pathways are not functional in these cells^20,21^. Consequently, rates of viral evolution observed during serial passage in cell cultures can be influenced by the initial choice of the viral strain used.

Notably, we found a high level of parallel evolution between viruses passaged in mosquito cells. Indeed, more than 35% of the mutations detected were shared between at least two passaged viruses. This phenomenon was common to viruses passaged in the same cell line, especially in both *Ae. aegypti* cell lines, probably in relation to the difficulties experienced to adapt our CHIKV strain in these cells. This typical phenomenon was previously observed during serial passage of arboviruses and in response to strong pressure selection (i.e. antimicrobial treatment, increased incubation temperature)^10,11,13,14,33,34^.

Among these shared mutations, we found 4 deletion mutations in regions encoding structural proteins and the junction region between the non-structural and structural ORFs. One deletion (8563_8568del) was previously found during serial passage in C6/36 cells^15^. Other amino-acid deletion mutations were detected in the hypervariable domain of the nsP3 protein^25,35^. The potential fitness effect of these deletion mutations in circulating CHIKV strains remains unclear^35^. However, the fact that we found no deletion during more than 300 serial passages in mammalian cells suggests that they confer fitness advantage in mosquito cells (previous published^15^ and personal data). To test this hypothesis, viruses passaged in mosquito cells and that harbored deletion mutations were passaged in mammalian cells. After only 10 serial passages, all the mutation deletions had disappeared. In addition, we observed that a large majority of the shared substitutions initially present also disappeared during serial passage in mammalian cells. Altogether, these results suggest that acquisition of deletion mutations constitutes an important mechanism for adaptation to mosquito cells and that a substantial proportion of the substitutions detected during serial passage in mosquito cells are vector-specific.

During serial passage in mosquito cells, we detected a high proportion of non-synonymous mutations many of which were located in specific mutational hot spots. The first is located in the X-domain, the N-terminal globular domain of the nsP3 region which is highly evolutionary conserved. This domain exhibits ADP-ribose 1″-phosphate phosphatase activity and contains ADP-ribose/poly(ADP-ribose) binding sites^36^. Two other highly variable domains were detected in the E2 and E1 proteins. These regions contained several *Ae. albopictus*-adaptative mutations involved in the multi-step process of vector switching^23,37^. This suggests that modifications in these regions are critical during adaptation to mosquito cells. Moreover, the mutational hot spot located in the E1 protein could be linked with the reversion of the E1-A226V adaptative mutations that we observed during all serial passages in mosquito cells. This confirms that this adaptative mutation does not provide a significant fitness advantage for replication in vector cells. Surprisingly, the distribution of non-synonymous mutations in our experiments and observed during evolution of ECSA CHIKV strains were very similar, suggesting that adaptation to mosquito cells is a significant driver of evolution of CHIKV during its natural life cycle.

We found that synonymous mutations detected in mosquito cells are not randomly distributed along the complete genome with a global distribution different from that observed for non-synonymous mutations. This highlights that a substantial proportion of synonymous mutations detected are not neutral and that specific constraints are applied to synonymous sites^38–40^. In particular, mutational hot spots of synonymous mutations were detected in regions encoding the N-terminal domain of the nsP1 and the 6K peptide which contain RNA secondary structures involve respectively in the initiation of viral genome replication and ribosomal frameshift^41,42^. On the other hand, even though the distribution of synonymous mutations did not match with the random distribution of the variable synonymous sites observed during evolution of ECSA CHIKV strains, an unexpected high number of common variables synonymous sites was found, suggesting the existence of vector-specific selection pressure acting at synonymous sites.

In conclusion, our results show that rates of viral evolution observed during serial passage *in cellulo* are affected by the initial choice of the cell line and the viral strain used and thereby cannot be extrapolated to explain what is happening *in vivo*. In addition, we provide here key features of the mutations involved in specific adaptation to mosquito cells.

## Electronic supplementary material

Supplementary information

## Data Availability

All data generated or analysed during this study are included in this published article (and its Supplementary Information files).
