# Supplementary material for: Evolution of Chikungunya virus in mosquito cells
Source: Sci Rep. 2018 Nov 1;8:16175. doi: 10.1038/s41598-018-34561-x (PMC6212429; doi:10.1038/s41598-018-34561-x)
Supplement: Supplementary file 1 — Supplementary information [file 41598_2018_34561_MOESM1_ESM.pdf]

Evolution of Chikungunya virus in mosquito cells

Souand Mohamed Ali[1], Abdennour Amroun[1], Xavier de Lamballerie[1], Antoine Nougairède[1]

Affiliations

[1]: UMR "Émergence des Pathologies Virales" (EPV: Aix-Marseille Univ – IRD 190 – Inserm 1207 – EHESP – IHU Méditerranée Infection), Marseille, France

Supplemental data

Supplemental figure 1: Mutation emergence kinetic curves

Average number of substitutions detected in complete genome sequence during serial passages (only substitutions with a frequency ≥20% (**A**) or ≥75% (**B**) were considered). Passages 18 and 31 in C6/36 cells are the starting point of serial passages in AE and AA-A20 cells respectively.

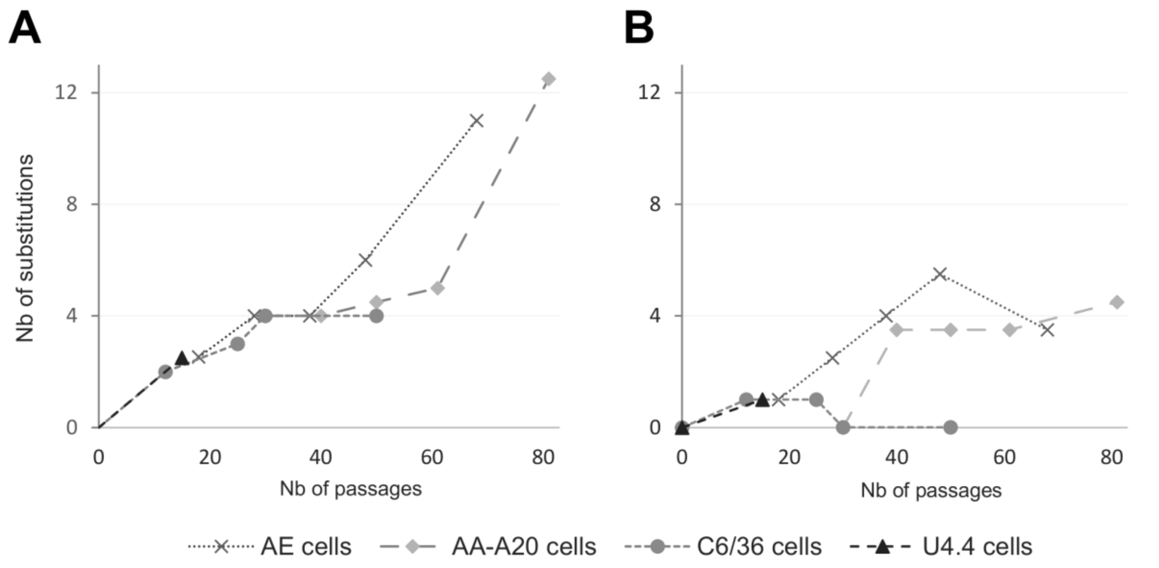

Supplemental figure 2: Characteristics of the substitutions detected per cell line

For each cell line, each variable nucleotide position (SNP site) was counted only once.

A: All the substitutions detected

B: Only substitutions with a frequency ≥20%

C: Only substitutions with a frequency ≤20%

The maximum frequency was considered when one substitution was detected more than once.

n: number of variable nucleotide position (SNP site) detected.

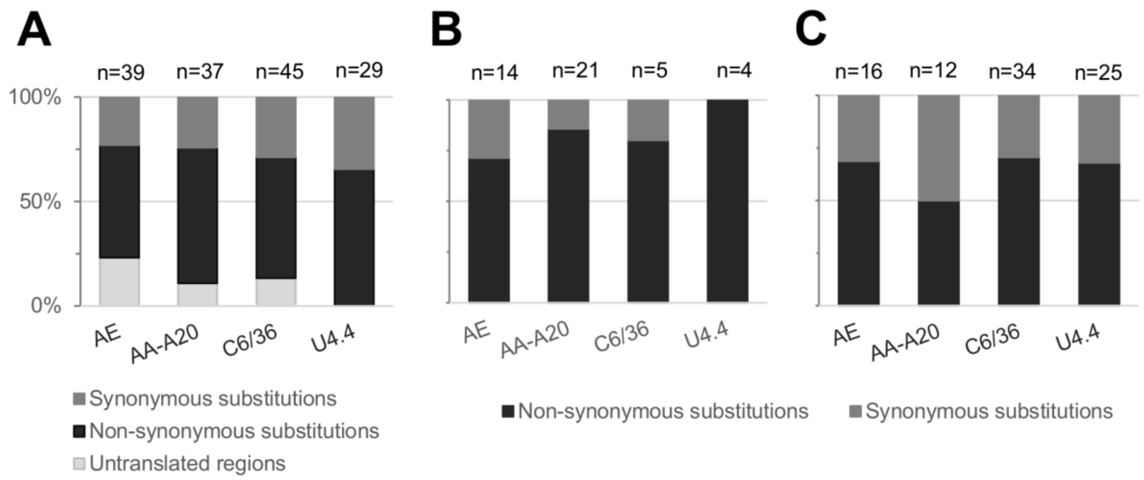

Supplemental table 1: Substitutions detected after 20 passages in C6/36 cells of a CHIKV strain pre-adapted to Vero cells

The strain was serially passaged 80 times in Vero cells (pre-adaptation) and then 20 times in duplicate in C6/36 cells. The complete genome sequence of the virus passaged 80 times in Vero cells was used as reference for substitution analysis.

Nucleotide position	Region	Nucleotide change	AA change	Virus harbouring the mutation	
				Duplicate #1 (substitution frequency)	Duplicate #2 (substitution frequency)
202	nsP1	G→C	A	0.25	1.00
764	nsP1	G→A	G→R	0.25	-
3703	nsP2	A→G		0.25	-
4139	nsP3	G→A	A→R	0.25	-
4509	nsP3	A→G	N→G	0.50	1.00
4662	nsP3	C→U	A→V	0.25	-
6061	nsP4	C→U	-	-	1.00
6104	nsP4	G→A	A→U	-	1.00
6272	nsP4	A→C	U→P	-	1.00
6279	nsP4	G→A	R→K	-	0.25
6407	nsP4	U→G	I→V	1.00	-
7113	nsP4	G→A	C→Y	0.75	-
8653	E2	A→G	R→G	-	0.25
8655	E2	A→U	-	0.25	-
8660	E2	A→G	E→G	0.75	-
8704	E2	G→A	G→R	0.25	-
9293	E2	G→A	R→Q	0.75	-
10670	E1	U→C	V→A	1.00	0.50

Supplemental table 2: Transitory substitutions detected during serial passage in mosquito cells

Nucleotide position	Region	Nucleotide change	AA change	Virus(es) harbouring the transitory mutation	
				Maximum substitution frequency between 20% and 75%	Maximum substitution frequency ≥ 75%
4151	nsP3	C→U	R→C	-	AE-2, A20-2
4295	nsP3	U→C	S→P	-	A20-2
6407	nsp4	U→G	I→V	-	A20-2
7502	junction	G→A	-	-	A20-2
7522	junction	U→C	-	-	AE-2
8702	E2	U→C	I→T	A20-1	AE-2, A20-2
8738	E2	A→C	K→T	A20-1	-
9777	E2	U→C	-	-	AE-2, A20-2

Supplemental table 3: Follow-up of frequency of shared mutations detected in mosquito cells after 10 passages in Vero cells

Two viruses already passaged in mosquito cells (C6-1 passage 31; U4-1 passage 15) were then serially passaged 10 times in duplicate in Vero cells. The frequency of the shared mutations present before serial passage in Vero cells was followed using complete genome sequences established after 10 passages in Vero cells.

Viruses	Nucleotide position	Region	Nucleotide change	AA change	Frequency of shared mutation before passages in Vero cells	Frequency of shared mutation after 10 passages in Vero cells	
						Duplicate #1	Duplicate #2
C6-1 P31	22	5'UTR	A→U	-	0.62	0.40	0.53
	7189	nsP4	G→U	-	0.03	0.00	0.00
	8563-8568	E2	Del-GUCUAU	Del-VY	0.75	0.00	0.00
	9037-9039	E2	Del-GAG	Del-E	0.04	0.00	0.00
	10670	E1	U→C	V→A	0.09	0.00	0.00
U4-1 P15	4151	nsP3	C→U	R→C	0.78	0.00	0.00
	4587	nsP3	U→C	I→U	0.03	0.63	0.00
	7189	nsP4	G→U	-	0.02	0.00	0.00
	9037-9039	E2	Del-GAG	Del-E	1.00	0.00	0.00
	10670	E1	U→C	V→A	0.26	0.00	0.00

Supplemental table 4: Mutations detected during serial passage in mosquito cells

Nucleotide position	passage	Region	Nucleotide change	AA change	Substitution frequency	Virus harboring the mutation
20	12	5'UTR	G→A	-	0,33	C6-2
20	25	5'UTR	G→A	-	0,11	C6-2
22	68	5'UTR	A→U	-	1,00	AE-1
22	28	5'UTR	A→U	-	1,00	AE-1
22	38	5'UTR	A→U	-	1,00	AE-1
22	48	5'UTR	A→U	-	1,00	AE-1
22	68	5'UTR	A→U	-	1,00	AE-2
22	41	5'UTR	A→U	-	1,00	A20-1
22	31	5'UTR	A→U	-	0,62	C6-1
22	50	5'UTR	A→U	-	0,07	C6-1
23	38	5'UTR	G→A	-	0,06	AE-2
24	25	5'UTR	C→U	-	0,02	C6-2
28	81	5'UTR	C→U	-	0,07	A20-2
63	50	5'UTR	A→C	-	0,03	C6-1
103	25	nsP1	C→U	-	0,05	C6-2
202	68	nsP1	G→A	-	0,61	AE-1
202	68	nsP1	G→A	-	0,42	AE-2
226	68	nsP1	A→G	-	0,02	AE-2
226	68	nsP1	A→G	-	0,08	AE-1
319	50	nsP1	C→U	-	0,07	C6-1
442	81	nsP1	A→G	-	0,46	A20-1
442	81	nsP1	A→G	-	0,12	A20-2
504	81	nsP1	A→G	Q→R	0,03	A20-2
764	25	nsP1	G→A	G→R	0,03	C6-2
772	31	nsP1	G→A	-	0,02	C6-1
772	50	nsP1	G→A	-	0,05	C6-1
782	68	nsP1	U→C	C→R	0,35	AE-1
782	68	nsP1	U→C	C→R	0,34	AE-2
1320	81	nsP1	U→C	L→P	0,50	A20-1
1320	81	nsP1	U→C	L→P	0,91	A20-2
1636	15	nsP1	U→C	-	0,03	U4-2
1866	15	nsP2	U→C	V→A	0,06	U4-2
1976	81	nsP2	G→U	V→L	1,00	A20-1
1976	61	nsP2	G→U	V→L	1,00	A20-1
1976	81	nsP2	G→U	V→L	1,00	A20-2
1976	51	nsP2	G→U	V→L	1,00	A20-1
1988	25	nsP2	C→U	-	0,97	C6-2
1988	12	nsP2	C→U	-	0,97	C6-2
2850	31	nsP2	U→C	V→A	0,07	C6-1
2868	51	nsP2	A→G	K→R	0,05	A20-2
3040	68	nsP2	G→A	-	0,09	AE-1
3040	68	nsP2	G→A	-	0,09	AE-2
3179	12	nsP2	U→A	S→T	0,04	C6-2
3263	68	nsP2	G→A	V→M	0,02	AE-2
3306	15	nsP2	U→C	V→A	0,06	U4-2
3358	15	nsP2	A→U	-	0,05	U4-2
3380	15	nsP2	A→G	I→V	0,03	U4-2
3402	81	nsP2	U→C	F→S	0,03	A20-1
3402	81	nsP2	U→C	F→S	0,03	A20-2
3440	68	nsP2	G→A	V→M	0,37	AE-1
3440	68	nsP2	G→A	V→M	0,37	AE-2
3447	31	nsP2	C→U	T→I	0,13	C6-1
3897	50	nsP2	G→A	C→Y	0,09	C6-1
3897	31	nsP2	G→A	C→Y	0,07	C6-1
4020	50	nsP2	U→C	V→A	0,10	C6-1

4151	48	nsP3	C→U	R→C	1,00	AE-2
4151	28	nsP3	C→U	R→C	0,99	AE-2
4151	41	nsP3	C→U	R→C	1,00	A20-2
4151	38	nsP3	C→U	R→C	1,00	AE-2
4151	15	nsP3	C→U	R→C	0,78	U4-1
4151	15	nsP3	C→U	R→C	0,07	U4-2
4164	50	nsP3	G→A	G→D	0,06	C6-1
4167	68	nsP3	G→A	G→D	1,00	AE-1
4167	48	nsP3	G→A	G→D	1,00	AE-1
4167	50	nsP3	G→A	G→D	0,45	A20-1
4167	50	nsP3	G→A	G→D	0,47	A20-2
4167	68	nsP3	G→A	G→D	1,00	AE-2
4168	50	nsP3	C→U	-	0,04	C6-1
4292	15	nsP3	U→C	F→L	0,03	U4-2
4295	15	nsP3	U→C	S→P	0,11	U4-2
4295	61	nsP3	U→C	S→P	1,00	A20-2
4295	51	nsP3	U→C	S→P	1,00	A20-2
4416	50	nsP3	A→C	Y→S	0,03	C6-1
4454	15	nsP3	C→U	H→Y	0,05	U4-2
4505	28	nsP3	C→U	R→C	0,25	AE-1
4587	50	nsP3	U→C	I→T	0,20	C6-1
4587	15	nsP3	U→C	I→T	0,03	U4-1
4587	81	nsP3	U→C	I→T	1,00	A20-1
4587	61	nsP3	U→C	I→T	1,00	A20-1
4587	81	nsP3	U→C	I→T	1,00	A20-2
4587	51	nsP3	U→C	I→T	1,00	A20-1
4595	15	nsP3	G→A	D→N	0,06	U4-2
4772	15	nsP3	U→C	Y→H	0,06	U4-2
4854	15	nsP3	U→C	V→A	0,17	U4-1
4855	15	nsP3	C→U	-	0,04	U4-1
4927	81	nsP3	A→G	I→M	0,48	A20-1
4927	81	nsP3	A→G	I→M	0,51	A20-2
5082	68	nsP3	C→U	T →I	0,03	AE-1
5160	81	nsP3	C→U	A→V	1,00	A20-1
5160	61	nsP3	C→U	A→V	1,00	A20-1
5160	81	nsP3	C→U	A→V	1,00	A20-2
5160	51	nsP3	C→U	A→V	1,00	A20-1
5317	68	nsP3	U→C	-	0,64	AE-1
5317	48	nsP3	U→C	-	1,00	AE-1
5317	68	nsP3	U→C	-	0,62	AE-2
5376	15	nsP3	U→C	L→P	0,04	U4-2
5646	25	nsP3	G→U	R→I	0,13	C6-2
5705	50	nsP4	C→A	Q→K	0,17	C6-1
5709	51	nsP4	A→G	K→R	0,23	A20-1
5715	50	nsP4	U→C	V→A	0,08	C6-1
5776	81	nsP4	A→U	-	0,08	A20-1
5776	81	nsP4	A→U	-	0,07	A20-2
5903	68	nsP4	C→U	-	0,02	AE-2
6105	50	nsP4	C→A	A→E	0,08	C6-1
6158	68	nsP4	A→U	N→Y	0,37	AE-1
6158	68	nsP4	A→U	N→Y	0,36	AE-2
6407	61	nsP4	U→G	I→V	1,00	A20-2
6407	51	nsP4	A→G	I→V	1,00	A20-2
6497	25	nsP4	C→U	-	0,03	C6-2
6955	61	nsP4	A→G	-	0,25	A20-1
6955	81	nsP4	A→G	-	0,05	A20-1
6955	81	nsP4	A→G	-	0,04	A20-2
6955	51	nsP4	A→G	-	0,25	A20-1
6970	81	nsP4	U→A	-	0,03	A20-1
6970	81	nsP4	U→A	-	0,03	A20-2
7189	31	nsP4	G→U	-	0,03	C6-1
7189	25	nsP4	G→U	-	0,04	C6-2
7189	15	nsP4	G→U	-	0,02	U4-1
7189	12	nsP4	G→U	-	0,03	C6-2

7416	68	nsP4	U→A	M→K	0,63	AE-1
7416	48	nsP4	U→A	M→K	0,75	AE-1
7416	48	nsP4	U→A	M→K	0,50	AE-2
7416	81	nsP4	U→A	M→K	0,51	A20-1
7416	81	nsP4	U→A	M→K	0,50	A20-2
7416	68	nsP4	U→A	M→K	0,61	AE-2
7416	38	nsP4	U→A	M→K	0,02	AE-2
7502	68	junction	G→A	-	0,08	AE-1
7502	41	Junction	G→A	-	1,00	A20-2
7502	68	junction	G→A	-	0,08	AE-2
7519	68	junction	C→A	-	0,38	AE-1
7519	68	junction	C→A	-	0,36	AE-2
7520	68	junction	U→A	-	0,03	AE-2
7522	68	junction	U→C	-	0,04	AE-1
7522	48	junction	U→C	-	1,00	AE-2
7522	68	junction	U→C	-	0,05	AE-2
7522	38	junction	U→C	-	0,88	AE-2
7540	50	junction	C→U	-	0,09	C6-1
7540	31	junction	C→U	-	0,07	C6-1
8018	15	C	A→C	K→T	0,06	U4-2
8549	51	E2	A→G	K→R	0,04	A20-1
8549	15	E2	A→G	K→R	0,25	U4-1
8564	15	E2	U→C	V→A	0,03	U4-1
8566	50	E2	U→C	Y→H	0,48	C6-1
8566	25	E2	U→C	Y→H	0,25	C6-1
8566	31	E2	U→C	Y→H	0,70	C6-1
8570	12	E2	A→C	K→T	0,46	C6-2
8702	61	E2	U→C	I→T	0,25	A20-1
8702	48	E2	U→C	I→T	1,00	AE-2
8702	28	E2	U→C	I→T	0,75	AE-2
8702	41	E2	U→C	I→T	1,00	A20-2
8702	38	E2	U→C	I→T	1,00	AE-2
8738	81	E2	A→C	K→T	0,44	A20-1
8738	61	E2	A→C	K→T	0,50	A20-1
8738	81	E2	A→C	K→T	0,43	A20-2
8784	15	E2	G→A	-	0,05	U4-2
8836	50	E2	C→U	H→Y	0,04	C6-1
8920	25	E2	C→A	H→N	0,04	C6-2
8920	38	E2	C→A	H→N	0,03	AE-2
9019	68	E2	A→U	T→S	0,98	AE-1
9019	28	E2	A→U	T→S	0,50	AE-1
9019	38	E2	A→U	T→S	1,00	AE-1
9019	48	E2	A→U	T→S	1,00	AE-1
9019	41	E2	A→U	T→S	0,25	A20-1
9041	15	E2	U→C	I→T	0,02	U4-1
9044	25	E2	A→U	E→V	0,08	C6-2
9054	68	E2	G→C	M→I	0,03	AE-2
9055	68	E2	C→G	-	0,03	AE-2
9058	25	E2	C→A	P→T	0,05	C6-2
9064	68	E2	A→G	T→A	0,03	AE-1
9064	68	E2	A→G	T→A	0,04	AE-2
9157	25	E2	U→G	S→A	0,02	C6-2
9203	50	E2	A→U	K→M	0,04	C6-1
9253	25	E2	A→G	N→D	0,10	C6-2
9254	25	E2	A→U	N→I	0,17	C6-2
9271	25	E2	C→U	R→C	0,13	C6-2
9272	25	E2	G→A	R→H	0,13	C6-2
9297	25	E2	A→U	K→N	0,02	C6-2
9315	25	E2	U→C	I→T	0,10	C6-2
9311	15	E2	U→G	I→S	0,02	U4-1
9311	81	E2	U→G	I→S	0,51	A20-1
9311	81	E2	U→G	I→S	0,52	A20-2
9326	15	E2	C→U	A→V	0,05	U4-1
9328	15	E2	A→G	N→D	0,24	U4-1

9328	15	E2	A→G	N→D	0,06	U4-2
9624	15	E2	G→A	-	0,07	U4-1
9663	68	E2	C→G	-	0,02	AE-1
9669	15	E2	C→U	-	0,03	U4-1
9777	38	E2	U→C	-	0,98	AE-2
9777	48	E2	U→C	-	1,00	AE-2
9777	28	E2	U→C	-	0,74	AE-2
9777	41	E2	U→C	-	1,00	A20-2
9904	50	6K	C→U	-	0,06	C6-1
10038	51	E1	U→C	-	0,03	A20-2
10069	15	E1	C→U	P→S	0,05	U4-2
10101	15	E1	C→U	-	0,02	U4-2
10339	81	E1	A→G	T→A	0,51	A20-1
10339	81	E1	A→G	T→A	0,53	A20-2
10380	51	E1	A→G	-	0,07	A20-1
10445	81	E1	A→G	D→G	0,06	A20-1
10445	81	E1	A→G	D→G	0,07	A20-2
10449	81	E1	U→C	-	0,07	A20-1
10449	81	E1	U→C	-	0,08	A20-2
10532	68	E1	A→C	Y → S	0,02	AE-1
10547	68	E1	A→U	Y →F	0,02	AE-1
10548	68	E1	U→A	Y → Z	0,02	AE-1
10631	81	E1	U→C	V→A	0,12	A20-2
10631	68	E1	U→C	V→A	0,05	AE-2
10670	68	E1	U→C	V→A	0,91	AE-1
10670	28	E1	U→C	V→A	1,00	AE-1
10670	38	E1	U→C	V→A	1,00	AE-1
10670	48	E1	U→C	V→A	1,00	AE-1
10670	41	E1	U→C	V→A	1,00	A20-1
10670	61	E1	U→C	V→A	1,00	A20-1
10670	41	E1	U→C	V→A	1,00	A20-2
10670	61	E1	U→C	V→A	1,00	A20-2
10670	25	E1	U→C	V→A	0,50	C6-1
10670	81	E1	U→C	V→A	0,96	A20-1
10670	81	E1	U→C	V→A	1,00	A20-2
10670	48	E1	U→C	V→A	1,00	AE-2
10670	68	E1	U→C	V→A	0,95	AE-2
10670	12	E1	U→C	V→A	0,95	C6-2
10670	50	E1	U→C	V→A	0,68	C6-1
10670	15	E1	U→C	V→A	0,26	U4-1
10670	25	E1	U→C	V→A	0,99	C6-2
10670	28	E1	U→C	V→A	0,98	AE-2
10670	51	E1	U→C	V→A	1,00	A20-1
10670	38	E1	U→C	V→A	0,99	AE-2
10670	51	E1	U→C	V→A	0,98	A20-2
10670	15	E1	U→C	V→A	0,84	U4-2
10670	31	E1	U→C	V→A	0,09	C6-1
10719	68	E1	U→C	-	0,18	AE-2
10719	68	E1	U→C	-	0,28	AE-1
10765	38	E1	G→C	G→R	0,02	AE-2
10778	81	E1	C→U	A→V	0,31	A20-1
10778	81	E1	C→U	A→V	0,31	A20-2
10809	25	E1	C→U	-	0,09	C6-2
10943	50	E1	U→C	I→T	0,07	C6-1
10962	68	E1	C→U	-	0,07	AE-1
10962	51	E1	C→U	-	0,03	A20-2
11008	25	E1	A→G	I→V	0,25	C6-2
11008	12	E1	A→G	I→V	0,04	C6-2
11557	68	3'UTR	C→A	-	0,02	AE-1
11652	25	3'UTR	C→U	-	0,66	C6-2
11652	12	3'UTR	C→U	-	0,04	C6-2
11667	68	3'UTR	A→U	-	0,11	AE-1
11667	81	3'UTR	A→U	-	0,07	A20-2
11667	68	3'UTR	A→U	-	0,16	AE-2

11731	68	3'UTR	A→U	-	0,03	AE-2
7512-26	48	Junction	DEL CAGCUACCUAUUUG	-	1,00	AE-1
7512-26	38	Junction	DEL CAGCUACCUAUUUG	-	1,00	AE-2
7512-26	81	Junction	DEL CAGCUACCUAUUUG	-	1,00	A20-1
7512-26	51	Junction	DEL CAGCUACCUAUUUG	-	1,00	A20-2
8392-94	68	E3	DEL-UUC	Del F	0,04	AE-1
8392-94	68	E3	DEL-UUC	Del F	0,05	AE-2
8563-68	31	E2	DEL-GUCUAU	Del VY	0,75	C6-1
8563-68	50	E2	DEL-GUCUAU	Del VY	0,05	C6-1
9037-39	28	E2	DEL-GAG	Del E	1,00	AE-1
9037-39	38	E2	DEL-GAG	Del E	1,00	AE-1
9037-39	58	E2	DEL-GAG	Del E	1,00	AE-1
9037-39	68	E2	DEL-GAG	Del E	1,00	AE-1
9037-39	28	E2	DEL-GAG	Del E	1,00	AE-2
9037-39	38	E2	DEL-GAG	Del E	1,00	AE-2
9037-39	58	E2	DEL-GAG	Del E	1,00	AE-2
9037-39	68	E2	DEL-GAG	Del E	1,00	AE-2
9037-39	41	E2	DEL-GAG	Del E	1,00	A20-1
9037-39	51	E2	DEL-GAG	Del E	1,00	A20-1
9037-39	61	E2	DEL-GAG	Del E	1,00	A20-1
9037-39	81	E2	DEL-GAG	Del E	1,00	A20-1
9037-39	41	E2	DEL-GAG	Del E	1,00	A20-2
9037-39	51	E2	DEL-GAG	Del E	1,00	A20-2
9037-39	61	E2	DEL-GAG	Del E	1,00	A20-2
9037-39	81	E2	DEL-GAG	DEL E	1,00	A20-2
9037-39	31	E2	DEL-GAG	DEL E	0,04	C6-1
9037-39	50	E2	DEL-GAG	DEL E	0,23	C6-1
9037-39	15	E2	DEL-GAG	DEL E	1,00	U4-1
9037-39	15	E2	DEL-GAG	DEL E	1,00	U4-2

Supplemental table 5: Summary of sequencing data used in this study

Passages in Vero cells are not represented in this table. Virus name and passage number are represented. NGS means complete genome sequence obtained using NGS method. S means complete genome sequence obtained using Sanger method.

**Virus**	**p10**	**p12**	**p15**	**p18**	**p25**	**p28**	**p31**	**p38**	**p41**	**p48**	**p50**	**p51**	**p61**	**p68**	**p81**
**C6-1**	S	-	-	S	S	-	NGS	-	-	-	NGS	-	-	-	-
**C6-2**	-	NGS	-	-	NGS	-	-	-	-	-	-	-	-	-	-
**U4-1**	-	-	NGS	-	-	-	-	-	-	-	-	-	-	-	-
**U4-2**	-	-	NGS	-	-	-	-	-	-	-	-	-	-	-	-
**A20-1**								-	NGS	-	-	NGS	NGS	-	NGS
**A20-2**								-	NGS	-	-	NGS	NGS	-	NGS
**AE-1**					-	NGS	-	NGS	-	S	-	-	-	NGS	-
**AE-2**					-	NGS	-	NGS	-	S	-	-	-	NGS	-

Supplemental table 6: Primers used for Next Generation sequencing

Sequence	Forward/Reverse	Nt position
ATGGCTGCGTGAGACACAC	Forward 1	1-19
GACCTTCGATACATTCCAAA	Forward 2	3079-3098
ACACACTACAGAATGTACTGGCA	Forward 3	6243-6265
CAGCACCGTGTACGATTACTGG	Forward 4	8804-8825
TGAGACCACTGCCTATCATTTA	Reverse 1	3162-3183
GAACCTATCCATTGGTACATC	Reverse 2	6491-6511
CGTACATGAGTGACTAATCTTCCT	Reverse 3	8893-8916
GTTCGGAGAATCGTGGAAGAGTTC	Reverse 4	11747-11770

Supplemental table 7: Primers and probes used for the real time RT-PCR assays

Sequence	Forward/Reverse/Probe	Nt position
TGACCGCCATTGTGTCATCGTTG	Foward	2631-2653
GACCTCGTATCCACGATAGTCA	Reverse	2788-2809
CTGGAGACCTCGTGTTAACGTGCTTCAG (5'FAM; 3'TAMRA)	Probe	2736-2763

Supplemental note 1: list of the CHIKV sequences extracted from GenBank used to determine the variability at 1st+2nd and 3rd codon positions

FJ445502 ; FJ445428 ; FJ513657 ; AB455494 ; AB455493 ; KP003813 ; KP003812 ; KP003810 ; KP003810 ; KP003808 ; KP003807 ; JN558836 ; JN558835 ; JN558834 ; JF274082 ; JQ067624 ; HM045822 ; HM045812 ; HM045809 ; HM045799 ; HM045794 ; HM045792; HM045784; HQ456253; AF490259 ; GU301781 ; GU301780 ; GU301779 ; EU037962 ; GQ905863 ; GU199352 ; GU199350 ; GU013529 ; FJ807899 ; FJ807898 ; FJ807896 ; GQ428215 ; GQ428214 ; GQ428212 ; GQ428211 ; GQ428210 ; FJ959103 ; FJ000065 ; FJ000062 ; EF210157 ; EF027134 ; AM258991
